# Detection of *Helicobacter pylori *in Oral Lesions

**DOI:** 10.5681/joddd.2013.037

**Published:** 2013-12-18

**Authors:** Soussan Irani, Alireza Monsef Esfahani, Farahnaz Bidari Zerehpoush

**Affiliations:** ^1^Assistant Professor, Department of Oral & Maxillofacial Pathologisty, Dental Faculty, Hamadan University of Medical Sciences, Hamadan, Iran.; ^2^Associate Professor, Anatomical Pathologist, Department of Pathology, Medical School, Hamadan University of Medical Sciences, Hamadan, Iran; Assistant Professor, Anatomical Pathologist, Department of Pathology, Medical School, Shahid Beheshti University of Medical Sciences, HakimLoghman Hospital, Tehran, Iran. ^3^

**Keywords:** *Helicobacter pylori*, oral ulcer, oral squamous cell carcinoma, oral lymphoma

## Abstract

***Background and aims. **Helicobacter pylori* is a microaerophilic gram-negative spiral organism. It is recognized as the etiologic factor for peptic ulcers, gastric adenocarcinoma and gastric lymphoma. Recently, it has been isolated from dental plaque and the dorsum of the tongue. This study was designed to assess the association between *H. pylori *and oral lesions such as ulcerative/inflammatory lesions, squamous cell carcinoma (SCC) and primary lymphoma.

***Materials and methods.*** A total of 228 biopsies diagnosed as oral ulcerative/inflammatory lesions, oral squamous cell carcinoma (OSCC) and oral primary lymphoma were selected from the archives of the Pathology Department. Thirty-two samples that were diagnosed as being without any pathological changes were selected as the control group. All the paraffin blocks were cut for hematoxylin and eosin staining to confirm the diagnoses and then the samples were prepared for immunohistochemistry staining. Data were collected and analyzed.

***Results.*** Chi-squared test showed significant differences between the frequency of* H. pylori *positivity in normal tissue and the lesions were examined (P=0.000). In addition, there was a statistically significant difference between the lesions examined (P=0.042). Chi-squared test showed significant differences between *H. pylori *positivity and different tissue types except inside the muscle layer as follows: in epithelium and in lamina propria (P=0.000), inside the blood vessels (P=0.003), inside the salivary gland duct (P=0.036), and muscle layer (P=0.122).

***Conclusion.*** There might be a relation between the presence of* H. pylori *and oral lesions. Therefore, early detection and eradication of* H. pylori *in high-risk patients are suggested.

## Introduction

The majority of head and neck cancer cases are related to tobacco use and heavy alcohol consumption.^1^ Other possible risk factors include viral infections,^2^ infection with *Candida* species and poor oral hygiene.^3^ A number of bacterial species are associated with different cancers.^4^ Increasing evidence shows the association of bacteria with some oral cancers.^5,6^ There is also a great diversity between different biological surfaces in the oral cavity for colonization of different bacterial species. For example, the salivary microbiota is mostly similar to that of the dorsal and lateral surfaces of the tongue but supragingival bacteria colonization is different from the microbiota on the oral soft tissue surfaces and in saliva.^7^

*Helicobacter pylori (H. pylori) *is a microaerophilic gram-negative spiral organism. In 1983, *H. pylori *was isolated for the first time by Marshall and Warren from human gastric biopsy specimens.^8^Different studies have revealed that* H. pylori* can be isolated from the oral cavity, dental plaque (supragingival and subgingival plaque), dorsum of the tongue and salivary secretions.^9-12^There are conflicting reports about the presence of* H. pylori* in the oral cavity and dental plaque. Wide variations in the prevalence of *H. pylori *in the oral cavity are partly due to employing different detection methods. For example, in a study by Butt et al, using urease test and cytology, *H. pylori *was detected in 100% and 88% of dental plaque samples, respectively.^13^In another study,* H. pylori *was detected in the saliva of 54.1% and in dental pockets in 48.3% of examined cases,^11^ and was considered a resident of the oral cavity. However, Chitsaziet al detected *H. pylori* in 34.1% of dental plaque samples.^14^

In addition, the presence of *H. pylori *was reported by Silva et al, using PCR, in 11.3% of supragingival plaque samples with or without periodontal diseases.^15^In a study, Mravak-Stipetićet al detected *H. pylori *in 13.04% of patients with different oral lesions.^16^ In another study on head and neck malignant and premalignant conditions, *H. pylori* was detected in 62.2% of cases.^17^*H. pylori* exists in high prevalence in the saliva and may be transmitted orally or via the fecal-oral route.^18^

The association of* H. pylori* with the pathogenesis of peptic and duodenal ulcers, gastric adenocarcinoma and low-grade B-cell mucosa-associated lymphoid tissue lymphoma has also been proven.^19,20^

*H. pylori* might have a role in the pathogenesis of oral lesions, e.g. ulcers, carcinomas and lymphomas. To assess this association, this study was designed to detect* H. pylori* in oral lesions including ulcerative/inflammatory lesions, squamous cell carcinoma (SCC) and primary lymphoma.

## Materials and Methods

A total of 228 biopsies diagnosed as ulcerative/inflammatory lesions, oral squamous cell carcinoma (OSCC) and oral primary lymphoma were selected from the archives of the Pathology Department. Thirty-two tissue samples taken from different areas of the oral cavity for other purposes, such as crown lengthening, and also samples with pathology reports stating “without significant pathological changes” were selected as the control group.

All the paraffin blocks were cut for H&E staining to confirm the diagnoses and then the samples were prepared for the immunohistochemistry (IHC) staining.

**Figure 1.**
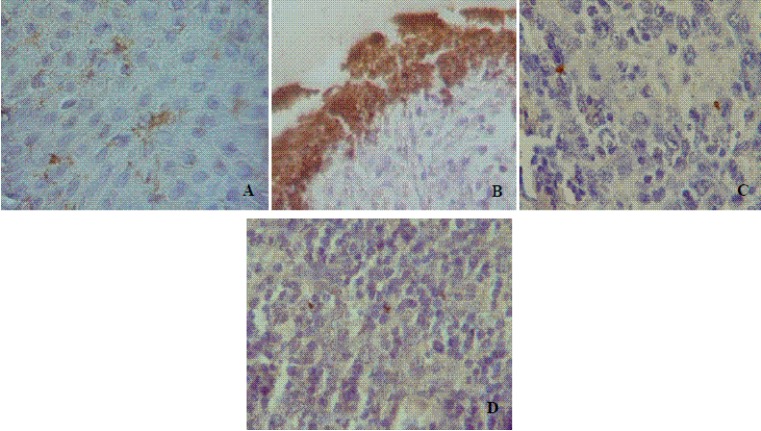
Sections of oral mucosa immunostained with *H. pylori* antibody. A) In the normal epithelium. B) Over the ulcer. C) In squamous cell carcinoma section. D) Primary lymphoma (×1000).

**Figure 2.**
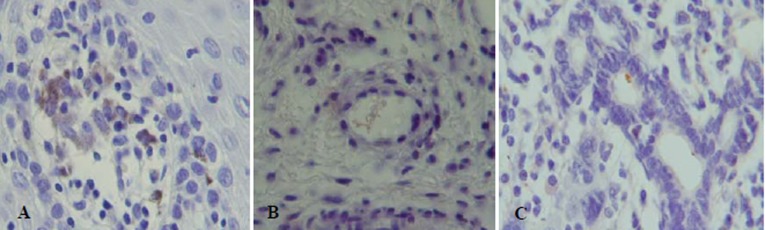
The coccoid and irregular forms of* H. pylori. *A) Within the lamina propria. Note also* H. pylori* in macro-phages. B) Inside the blood vessel. C) Inside the salivary duct (×1000).

Briefly, 4-μm-thick sections of paraffin-embedded formalin-fixed specimens were cut. The slides were deparaffinized, rehydrated and pre-treated with trypsin for 40 minutes at 37°C according to manufacturer’s instructions (Novocastra, UK). The endogenous peroxidase activity was blocked, followed by incubation with lyophilized rabbit polyclonal antibody (Novocastra) at a dilution of 1:20 for 1 hour. DAB was used to visualize the complex. Then, the sections were counterstained with hematoxylin and mounted. *H. pylori*-positive and -negative human gastric samples were used as positive and negative controls, respectively(Figures 1 and 2).

Statistical analysis was performed with SPSS 11.0.1 using chi-squared test. Statistical significance between the groups was set at P<0.05.

## Results

In this study, there were 141 males (54.2%) and 119 females (45.8%). In general, the ages of the patients ranged from 7 to 80 years, with a mean age of 43.18 years. Demographic data of the samples are shown in Table 1.

**Table 1:** Demographic characteristics of samples

Study group	No. of cases	Male	Female	Median age (years)	Range of age
Normal tissue	32(12.3%)	9	23	39.6	7-78
Ulcerative/Inflammatory lesion	117(45%)	75	42	38.9	7-80
Squamous cell carcinoma	83(31.9%)	39	44	50.9	31-75
Lymphoma	28(10.7%)	18	10	42.3	34-68
Total	260	141	119	43.18	7-80

Table 2 shows the presence of *H. pylori* in different areas of the oral cavity. According to Table 2, *H. pylori* positivity was mostly found in the tonsils and tongue, with 43 (16.5%) and 42 (16.1%) cases, respectively.* H. pylori *negativity was mostly found in the tongue, with 17 (6.5%) cases, followed by the buccal mucosa and oropharynx, with nine (3.4%) cases each. According to Table 2, most of the tonsil and tongue *H. pylori* positivity was found in ulcerative/inflammatory lesions, with 37 cases (14.2%) and 26 cases (10%), respectively. On the other hand, most of the* H. pylori*-positive SCC samples were found in the soft palate and oropharynx, with 14 cases (5.3%) each. The buccal mucosa was the most common site for *H. pylori* positivity in lymphoma, with six cases (2.3%).

**Table 2:** Summary of *H. pylori* detection (in numbers) in different regions

	Normal tissue	Ulcerative/Inflammatory lesion	SCC	Lymphoma
*H. pylori* status	+	-	+	-	+	-	+	-
Buccal mucosa	1	4	3	2	6	2	6	1
Floor of mouth	1	3	1	1	4	2	0	0
Tongue	1	3	26	13	13	0	2	1
Tonsil	1	2	37	2	2	0	3	2
Retromolar area	1	2	0	0	8	1	1	1
Gingiva	2	2	5	4	12	2	0	0
Vestibule	1	1	8	3	0	0	1	1
Palate	1	1	2	4	8	2	1	1
Soft palate	2	1	0	0	14	4	3	4
Oropharynx	2	1	0	0	14	4	3	4
Total	12	20	85	32	69	14	17	11

Table 3shows that the highest frequency of *H. pylori* positivity was detected in ulcerative/inflammatory lesions in 85 (32.6%) cases, followed by OSCC in 69 (26.5%) cases. The highest frequency of* H. pylori *negativity was also seen in ulcerative/inflammatory lesions, with 32 cases (12.3%), followed by normal tissue, with 20 cases (7.6%).

**Table 3:** Frequency of *H. pylori* detection in different lesions

Type of Lesion	H. p Positive	H. p Negative	P
Normal tissue	12 4.6%	20 7.6%	All samples (0.000)
Ulcerative/Inflammatory lesions	85 32.6%	32 12.3%	All lesions (0.042)
SCC	69 26.5%	14 5.3%	
Lymphoma	17 6.5%	11 4.2%	
Total	183 70.4%	77 29.6%	

A summary of the presence of* H. pylori* in different tissue types is shown in Table 4. In all the lesions, *H. pylori* was mostly detected in the epithelium, with 181 cases (69.6%), followed by the lamina propria, with 86 cases (33.4%). In 19 (7.3%) cases,* H. pylori* was detected in blood vessels, in 11 cases (4.2%) in salivary gland ducts and in one case (0.3%) in the muscle layer of the tongue.

As shown in Table 4, *H. pylori* epithelial positivity was mostly detected in ulcerative/inflammatory lesions in 85 cases (22.3%), followed by SCC in 67 cases (25.7%). Invasion to the lamina propria was also mostly detected in ulcerative/inflammatory lesions in 35 cases (13.5%), followed by SCC in 32 cases (12.3%).

**Table 4:** Summary of *H. pylori *detection in different tissue types

Type of tissue Type of lesion	Epithelium	Lamina propria	Blood vessel	Salivary gland duct	Muscle layer
Normal tissue	12 (14.6%)	10 (3.8%)	2	0	0
Ulcerative/Inflammatory Lesions	85 (32.7%)	35 (13.4%)	7	9	1
SCC	67 (25.6%)	32 (12.3%)	7	1	0
Lymphoma	17 (6.5%)	10 (3.8%)	3	1	0

Chi-squared test showed significant differences between the frequency of* H. pylori* positivity in normal tissues and the lesions examined (P=0.000). In addition, there was a statistically significant difference between the lesions examined (P=0.042).

Chi-squared test showed significant differences between *H. pylori* positivity and different tissue types except for intramuscular layer as follows: in the epithelium and in lamina propria (P=0.000), inside the blood vessels (P=0.003), inside salivary gland ducts (P=0.036), and muscle layer (P=0.122).

## Discussion

In this study, the presence of* H. pylori *in normal oral tissues and oral lesions, ulcerative/inflammatory lesions, SCC and primary lymphoma were reviewed using IHC.

There are several methods to detect *H. pylori*. One of these is the urease test. But, in the oral cavity, there are other bacteria producing urease, including *Streptococcus *spp, *Haemophilus *spp and *Actinomyces *spp; therefore, it is hard to suggest that high urease activity in the oral cavity is indicative of the presence of *H. pylori*.^21^

In the stomach, culture technique has been considered “the gold standard.” However, contrary to the stomach, there are many other organisms in the oral cavity. Therefore, there is a possibility of other faster-growing organisms in the culture media.^21^ On the other hand, in the oral cavity, the organisms in coccoid forms are nonculturable; therefore, the prevalence of *H. pylori *may be underestimated.^22^Additionally, some previous studies have indicated that culture methods could very rarely isolate *H. pylori* from saliva. Some previous studies have shown that other microorganisms prevent *H. pylori* from growing in the culture media.^23,24^

Polymerase chain reaction (PCR) is another accurate method for detecting *H. pylori*; however, because different primers are used, the results are variable. In addition, due to false-positive results, partly due to the detection of cDNA from non-*H. pylori *organisms, the results are not reliable.^12,21,25^ In case of a low number of organisms in the specimen, false-negative results may also occur.^26^ On the other hand, in the oral cavity, there is a complexity of microflora; hence, the specificity and sensitivity of selected primers are another important issue.^10^ To increase the specificity of PCR and to avoid inhibitors,* H. pylori *should be separated from the contaminants.^27^ The other problem is that because* H. pylori* gene can be detected using PCR, it is not clear whether the gene found belongs to live bacteria or not.^21,28^ PCR detects the DNA of bacteria that are also not viable. PCR also detects small numbers of bacteria that may not have a significant impact on oral cavity infections.^11^ PCR assays for *H. pylori *have a wide cross-reactivity and are positive when other microorganisms contain those sequences.^29^Finally, it is difficult to find sufficient patients with OSCC and oral primary lymphoma within a reasonable time frame.

IHC is another method for detecting *H. pylori*. Ito et al used reverse transcriptase PCR to detect *H. pylori *DNA in the histologic sections and compared the results with those obtained using IHC. They found that IHC is specific but less sensitive than PCR.^30^

In the present study, firstly, due to IHC specificity for *H. Pylori *detection and, secondly, due to the decision to show the location of *H. pylori* inside the tissue as well as its invasion to the lamina propria, IHC was employed to detect *H. pylori*.

*H. pylori* was detected in different regions of the oral cavity, in descending order, as follows: dental plaque, 82.3%, gargles, 51.1% and mucosa of the dorsum of the tongue, 37.5%, suggesting that *H. pylori *settles in more than one site.^31^ The number of microorganisms varies from one site to another within the oral cavity and is not uniformly distributed in the mouth.^32^

Two mechanisms have been suggested for *H. pylori *pathogenesis. First, *H. pylori *interacts with surface epithelial cells, developing direct cell damage or producing pro-inflammatory mediators.^33-35^ Second, *H. pylori* reaches the underlying mucosa to stimulate an immune response, leading to the release of different cytokines and oxygen radicals that transform the chronic gastritis into gastroduodenal ulcers and gastric carcinoma.^36-38^ According to previous reports, *H. pylori* produces extracellular products that cause local and systemic immune responses, which can result in tissue damage.^39-41^ Previous studies on the gastric mucosa indicated the presence of *H. pylori *in the lamina propria, the intercellular space as well as in the gastric lumen.^42^*H. Pylori* was also detected inside the blood vessels, which may explain *H. pylori* bacteremia, resulting in a systemic response.^43^

Intercellular *H. pylori *was found in duodenal ulcer samples.^44,45^ In areas like an ulcerated epithelium, *H. pylori* gets serum factors and therefore becomes more invasive.^46^

*H. pylori* can be found within the oral epithelium, such as buccal mucosa and the tongue.^17,47^In the present study, one case of a normal tonsil and 37 cases of ulcerated/inflammatory tonsils showed *H. pylori* positivity. In a study on 23 samples from tonsil and adenoid tissues, *H. pylori *was detected in seven samples (30%, four tonsil tissues and three adenoid tissues).^48^

In the current study,* H. pylori *was detected in 4.6% of normal samples and in 32.7% of ulcerative/inflammatory lesions. In a study on oral ulcers, *H. pylori* was detected in six (20.7%) out of 29 cases, and all the positive samples were located in the buccal mucosa.^47^ In another study, Mravak-Stipetic et al, using PCR, detected *H. pylori* in four patients (12.5%) with recurrent aphthous ulcers. All the control samples were negative.^16^ In another study on recurrent aphthous ulcers, 71.9% of cases were positive for *H. pylori*.^49^Fritscher et al, studying 105 children and adolescents, found that 9.4% of 53 patients with recurrent aphthous stomatitis were positive for *H. Pylori*, and in the control group only 3.8% were positive. They did not find any statistically significant relationship between the presence of* H. Pylori *and recurrent aphthous stomatitis.^50^

In our series, 26.5% of SCCs and 6.5% of lymphomas showed *H. pylori* positivity. Rubin et al, working on 61 samples from head and neck malignant and premalignant conditions, detected *H. pylori* positivity in 16.3% of oral cavity samples.^17^In a study using swab samples of the oral mucosa and cancer lesion surfaces, no positive PCR results were obtained.^24^According to previous reports, oral cancer has a high risk of secondary primary tumors. Patients surviving a previous oral cancer have up to a 20-fold increased risk of developing a second primary oral cancer.^51,52^ Poor oral hygiene increases the risk of oral cancer.^53^

A recent study reported that 40% of 39 patients had viable *H. pylori* in their oral cavities despite *H. pylori* eradication. In addition, 56% of those without detectable *H. pylori* in the mouth before treatment had *H. pylori* in the oral cavity when re-examined after *H. pylori* eradication.^54^Presence of* H. pylori* in the oral cavity, even after treatment, might explain the development of secondary primary tumors. It has been shown that* H. pylori* can multiply not only in macrophages but also in dendritic cells and epithelial cells. Residency inside infected cells increases its resistance to antimicrobial treatment and protects it from humoral antibody attack.^16^These findings can explain treatment failure.

The presence of *H. pylori* in the stromal cell of the lamina propria, far from the epithelial basement membrane, indicates invasion.^55^ Several studies have shown* H. pylori *invasion into the lamina propria of gastric mucosa, which can be an important factor in the induction and development of gastric inflammation.^30,33,35,46^In the present investigation,* H. pylori* was found in the epithelial layer of normal tissues as well as lesions in 69.6% and in the lamina propria in 33.4%, which can be clear evidence for the invasion of the bacteria. In one case, bacteria were found in the deep muscle layers of the tongue. Petersen et al found that *H. pylori* is able to pass through the endothelial layer.^46^ In the current study, in 7.3% of cases *H. pylori* was seen in the vessels, and it was also found in the salivary ducts in two cases.

In the present study, *H. pylori* oral colonization was seen in both the coccoid and the spiral forms. There are some other studies detecting *H. pylori *in the coccoid form. Many investigations have described whole bacterial cells, mainly of coccoid forms. The coccoid form of *H. pylori* is viable, but is not culturable and increases as infection proceeds. The coccoid form is more resistant to antibiotics and can spread to infect other cells in the absence of a therapeutic concentration of antibiotic.^56^ Wang et al suggested that the coccoid form of *H. pylori* is viable and maintains the integrity of the nucleic acid contents and active protein synthesis.^57^ In addition, the coccoid form of the microorganism is able to synthesize DNA.^22^ The present study detected the coccoid form of *H. pylori*, which might be proof for its long-standing persistence in the oral cavity and might reveal the role of *H. pylori* in the pathogenesis of the oral disorders examined. In this study, colonization and irregularly shaped bacteria and irregular dense bodies were found. Spiral, coccoid and degenerative forms and also irregularly shaped bacteria were found in other studies using electron microscopy.^42^

In the present study, in patients’ files, there were no clinical reports for gastritis or other stomach disorders. Several studies support the hypothesis that the oral cavity is a reservoir for re-infection of the stomach.^21,58^ On the other hand, some other investigations have shown that presence of *H. pylori* in the oral cavity does not relate to gastric infection and that *H. pylori* can also be found in the oral cavity without any gastric infection.^14,32,54,59^ In a study carried out by Song et al, it was shown that *H. pylori* DNA sequences differed between oral samples and gastric samples within the same individual.^60^

In conclusion, it is suggested that there is a relation between the presence of* H. pylori* in the oral cavity and in the oral lesions. It seems likely that the presence of *H. pylori* might be a risk factor for the developing oral lesions, ulcers and cancers. Oral infection sources such as dental plaque must be controlled to decrease the prevalence of oral cancer. The oral flora might be a diagnostic tool to predict oral lesions, such as oral cancer. Early detection and eradication of *H. pylori* in the oral cavity, especially in high-risk patients such as tobacco users, alcohol consumers, any patients with a history of gastritis or with cancer development in relatives, might prevent its consequences.
